# Significant changes of CD4, FOXP3, CD25, and IL6 expression level in Iranian COVID-19 patients 

**Published:** 2020

**Authors:** Seyed Reza Mohebbi, Kaveh Baghaei, Mohammad Rostami-Nejad, Ehsan Nazemalhosseini Mojarad, Hamed Mirjalali, Abbas Yadegar, Nastaran Asri, Shahrokh Abdoulahi, Hamid Assadzadeh Aghdaei

**Affiliations:** 1 *Gastroenterology and Liver Diseases Research Center, Research Institute for Gastroenterology and Liver Diseases, Shahid Beheshti University of Medical Sciences, Tehran, Iran*; 2 *Basic and Molecular Epidemiology of Gastrointestinal Disorders Research Center, Research Institute for Gastroenterology and Liver Diseases, Shahid Beheshti University of Medical Sciences, Tehran, Iran*; 3 *Foodborne and Waterborne Diseases Research Center, Research Institute for Gastroenterology and Liver Diseases, Shahid Beheshti University of Medical Sciences, Tehran, Iran*

**Keywords:** COVID-19, SARS-CoV-2, Inflammatory responses, Treg, Interleukin 6

## Abstract

**Aim:**

Evaluating the expression level of CD4^+^ FoxP3^+^ CD25^+^ T cells and IL-6 in peripheral blood samples of hospitalized COVID-19 patients.

**Background:**

COVID-19 is an emerging disease with worldwide distribution. However, there is a little data about the correlation between the disease and the host immune responses.

**Methods:**

Whole blood samples of 30 COVID-19 patients and eight healthy people were collected during March to June 2020. Total RNA was extracted from the samples, cDNA synthesis was performed, and the expression level of targeted genes was evaluated using quantitative real-time PCR.

**Results:**

The expression level of CD4, CD25, and Foxp3 was significantly downregulated 5-, 2-, and 3-fold, respectively, among COVID-19 patients in comparison to healthy controls (*P*-value < 0.0001). The expression level of IL-6 represented almost 18-fold increase in COVID-19 patients compared to healthy controls.

**Conclusion:**

Our findings indicated the expression profile analysis of CD4^+^ FoxP3^+^ CD25^+^ T cells could be a potential marker for the assessment of severity of COVID-19 patients.

## Introduction

 Coronavirus disease 2019 (COVID-19) is caused by severe acute respiratory syndrome-coronavirus 2 (SARS-CoV-2) that leads to severe adult respiratory distress syndrome (ARDS). COVID-19 seems to have emerged in December 2019 in Wuhan, China, now known as a pandemic all over the world (1, 2). Clinical evidence shows that cytokine storm, due to releasing IL-6, IL-12 and tumor necrosis factor α (TNF-α), probably plays an important role in the pathogenesis of SARS-CoV-2 pneumonia (3). High levels of IL-6, known as the critical point in the pathogenesis of COVID-19, imply that blocking it using monoclonal antibodies such as tocilizumab (RoActemra, Roche) and sarilumab (Kevzara, Sanofi), may constitute a therapeutic strategy in management of COVID-19 patients (4-7).

In addition, decrease of CD4 and CD8 cells and increase of Th17 cell proportion seem to be the pathology of COVID-19 (8). Th17 lineage is a CD4^+^ T cell subset that provokes secretion of proinflammatory cytokines and protects hosts against microbial infections. This lineage also performs a crucial role in the pathogenesis of immune-related diseases. The altered ratio between regulatory T (Treg) cells generally suppresses or downregulates the induction and proliferation of effector T cells (9).

Identification of Treg cells was originally based on CD25 expression; however, CD25 is also expressed by activated effector T cells. Foxp3 is a specific well-known indicator of Treg cells, and CD4^+^ Foxp3^+^ CD25^+^ T cells are recognized as the main natural Treg (nTreg) population group (10).

**Table 1:** Primersused in this study

Genes	Primer Sequence
CD4	F:5'-ACATCAAGGTTCTGCCCAC-3' R:5'-TGGCAGGTCTTCTTCTCAC-3'
CD25	F:5'-ACTTCCTGCCTCGTCACAAC-3' R:5'-ACTCTTCCTCTGTCTCCGCT-3
FoxP3	F:5'-TCATCTGTGGCATCATCCG-3' R:5'-AGGAACTCTGGGAATGTGC-3'
IL-6	F:GATTCAATGAGGAGACTTGCC R:GGTCAGGGGTGGTTATTGC

Due to lack of sufficient information about the expression levels of Tregs and its role in regulation of IL-6 cascade in COVID-19 patients, this study aimed to evaluate the expression level of IL-6 and CD4^+^ Foxp3^+^ CD25^+^T cells in COVID-19 patients. 

## Methods

***Sampling***

Between March to June 2020, whole blood samples were collected of 30 patients who were diagnosed and confirmed for COVID-19, based on clinical manifestations, radiology tests (CT scan), and SARS-CoV-2 molecular detection. Mean age (mean + SD) of the studied patients were 59.67 + 17.22 (range = 67 years, 23 - 90). Whole blood samples of eight healthy people negative for COVID-19 were considered as the control group. Demographic data and history of symptoms were collected using a standard questionnaire. The Clinical Research Ethics Committee of Shahid Beheshti University of Medical Sciences and the Ethics Committee of Taleghani Hospital, Tehran, Iran (IR.SBMU.IRGLD.REC.1399.006) approved this survey.

***RNA extraction and cDNA synthesis***

Total RNA extraction was performed using the Total RNA extraction kit (Yekta Tajhiz Azma kit, Teheran, Iran) according to manufacture instructions. Purity and concentration of extracted RNA were evaluated by NanoDrop 1000 spectrophotometer (NanoDrop Technologies, Wilmington, DE, USA) and the integrity of RNA was ascertained using electrophoresis on a denaturing 1.5% agarose gel. After adjusting the RNA concentrations, cDNA synthesis was performed using the cDNA synthesis kit (TaKaRa kit, Otsu, Shiga, Japan). Synthesized cDNA was stored at -20°C until quantitative real-time PCR. 

**qReal-time PCR**

To evaluate the expression levels of targeted genes, qreal-time PCR was performed and implemented in a final amount of 20 μL containing 10 µL of 2X standard SYBR Premix Ex Taq™ kit (TaKaRa Bio Inc., Otsu, Japan), 5 μL of reverse-transcribed cDNA, and 5 pmol of each primer using Applied Biosystems 7500 Version 1 software (ABI, Foster City, CA, USA) under the following cycling status: 95°C for 5 s, 40 cycles of 95°C for 5 s, 60°C for 34 s, 95°C for 15 s, 60°C for 1 s and 60°C for 15s (Khatibi et al., 2018). The primers sequences and characteristics are shown in table 1 (Table 1). Normalization for samples amplification signals was performed using a glyceraldehyde 3-phosphate dehydrogenase (GAPDH) gene. Fold change assessment of gene expression was carried out by the 2^-ΔΔct^ method.

***Statistical analysis***

All findings were evaluated using Graph-pad Prism Version 5 software (San Diego, CA, USA). Non-parametric tests were utilized as the gathered data were non-normally distributed. Student t-test and the one-way ANOVA test were implemented. P-value < 0.05 was considered statistically significant.

## Results

**Figure 1:**
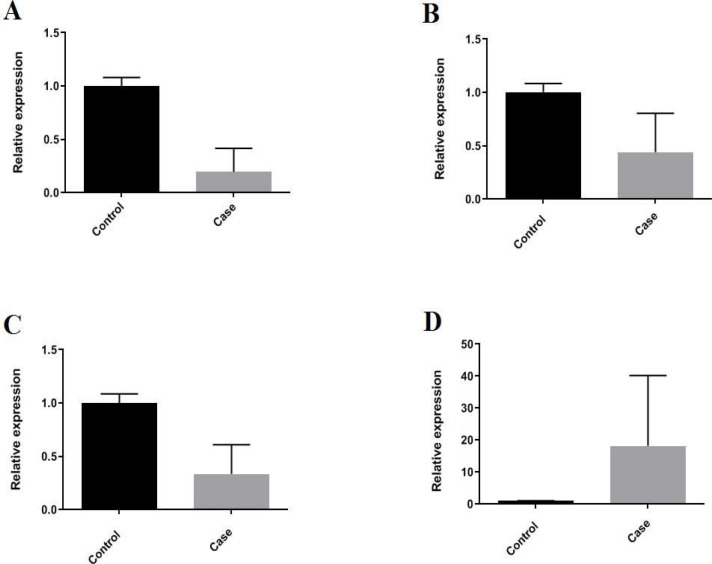
The relative expressions of A) TCD4, B) TCD25, C) FOXP3, and D) IL-6 showing increased level of IL-6 in contrast with decreased level of TCD4, TCD25, and FOXp3 in Covid-19 patients in comparison to healthy controls

All 30 symptomatic COVID-19 cases confirmed using PCR and a CT scan workup. The mean age of 59.67 + 17.22 (range = 67 years, 23 - 90), and 68% and 32% were male and female, respectively.

Based on the results, a statistically significant downregulation (5-fold changes) was observed in TCD4 among COVID-19 positive patients compared to healthy controls (P-value < 0.0001). As expected, the mRNA expression levels of CD25 and Foxp3 showed statistically significant downregulation as around 2 and 3-fold, respectively, in comparison to healthy controls (P-value < 0.0001). The expression levels of IL-6 represented almost 18-fold increase in COVID-19 patients compared to healthy controls (P-value<0.0001) (Figure 1).

## Discussion

This clinical cross-sectional study identified a decrease and increase of mRNA level in the T reg family and IL-6 in COVID-19 patients, respectively. Cytokine storm, due to elevated inflammatory cytokines, may play a major role in the pathology process of COVID-19 (4). 

Many studies have suggested that COVID-19 patient pathology damage records revealed tissue interstitial macrophage, and monocyte infiltrations in the lung, heart, and gastrointestinal mucosa which could be due to elevated inflammatory cytokines (8, 11). This cytokine release syndrome (CRS) assigns to a deregulated dispensation of proinflammatory mediators by an excessively activated immune system (13).

The results show a decrease in the mRNA expression levels of TCD4, CD25, and Foxp3 in COVID-19 patients. Similar to this finding, Qin et al reported that decreased regulatory T cells (12) are commonly seen in critically ill patients, suggesting dysregulated immune responses. Data from experimental study show IL-6 that arise in ARDS, may have contextual protective or exacerbating roles including severity of infection, survival and tissue remodeling (5-14).

In one systematic review and meta-analysis, Coomes et al. demonstrated that serum levels of IL-6 significantly elevate in severe COVID-19 disease cases. While inhibition of IL-6 with tocilizumab appears to be efficacious and safe in preliminary investigation, the results of several ongoing clinical trials should be wait to better define the role of tocilizumab in COVID-19 prior to routine clinical application (7). In another study Herold et al. documented that elevated interleukin-6 (IL-6) was strongly associated with the need for mechanical ventilation (15). Recently, Sadeghi and colleagues compared Treg and Th17 cells responses in COVID-19 intensive unit patients with healthy controls. They found a significant Treg cell number decline and also observed decrease in FoxP3 mRNA expression level in patients. In addition, lower levels of IL-10 and TGF-β cytokines were detected in patients in comparison to healthy controls (16). They concluded that increased levels of Th17, as well as reduced number of Treg cells and associated determinants such as FoxP3, IL10 and TGF-β might perform a leading role in elevating inflammation and pathogenesis of COVID-19. 

It seems that elevating IL-6 and Treg/Th17 imbalance and following dysregulated increase of pro-inflammatory cytokines and excessive systemic inflammation may lead to severe form of COVID-19 disease, serious lung injury, organ failure and death (17). 

In conclusion, there is limited data on Tregs in COVID-19 patients examined during the initial period of the COVID-19 pandemic. Our results showed that, Tregs are significantly decreased in COVID-19 patients, accompanied by increased IL-6. These data proposed that Treg cells may be a biomarker of severity and remark the probability that antibodies directing IL-6 might be able to moderate lymphocyte stability. Although this single study is not adequate to verify that Tregs play a role in COVID-19 development because of limited investigated subjects, we can, in future studies, achieve confirmation from in vitro investigation and animal trials.
